# Prevalence and nature of potential drug-drug interactions among hospitalized HIV patients presenting with suspected meningitis in Uganda

**DOI:** 10.1186/s12879-020-05296-w

**Published:** 2020-08-05

**Authors:** Prosperity C. Eneh, Katherine Huppler Hullsiek, Daniel Kiiza, Joshua Rhein, David B. Meya, David R. Boulware, Melanie R. Nicol

**Affiliations:** 1 Experimental and Clinical Pharmacology, University of Minnesota, Minneapolis, MN USA; 2 Biostatistics, University of Minnesota, Minneapolis, MN USA; 3 Infectious Disease Institute, Makerere University, Kampala, Uganda; 4 Infectious Diseases and International Medicine, University of Minnesota, Minneapolis, MN USA; 5 College of Health Sciences, Makerere University, Kampala, Uganda

**Keywords:** Drug-drug interactions, HIV, Suspected meningitis, Acute care, Cohort studies, DDIs

## Abstract

**Background:**

Management of co-infections including cryptococcal meningitis, tuberculosis and other opportunistic infections in persons living with HIV can lead to complex polypharmacotherapy and increased susceptibility to drug-drug interactions (DDIs). Here we characterize the frequency and types of potential DDIs (pDDIs) in hospitalized HIV patients presenting with suspected cryptococcal or tuberculous meningitis.

**Methods:**

In a retrospective review of three cryptococcal meningitis trials between 2010 and 2017 in Kampala, Uganda, medications received over hospitalization were documented and pDDI events were assessed. IBM Micromedex DRUGDEX® online drug reference system was used to identify and describe potential interactions as either contraindicated, major, moderate or minor. For antiretroviral DDIs, the Liverpool Drug Interactions Checker from the University of Liverpool was also used to further describe interactions observed.

**Results:**

In 1074 patients with suspected meningitis, pDDIs were present in 959 (overall prevalence = 89.3%) during the analyzed 30 day window. In total, 278 unique interacting drug pairs were identified resulting in 4582 pDDI events. Of all patients included in this study there was a mean frequency of 4.27 pDDIs per patient. Of the 4582 pDDI events, 11.3% contraindicated, 66.4% major, 17.4% moderate and 5% minor pDDIs were observed. Among all pDDIs identified, the most prevalent drugs implicated were fluconazole (58.4%), co-trimoxazole (25.7%), efavirenz (15.6%) and rifampin (10.2%). Twenty-one percent of the contraindicated pDDIs and 27% of the major ones involved an antiretroviral drug. Increased likelihood of QT interval prolongation was the most frequent potential clinical outcome. Dissonance in drug interaction checkers was noted requiring clinicians to consult more than one database in making clinical decisions about drug combinations.

**Conclusions:**

The overall prevalence of pDDIs in this population is high. An understanding of drug combinations likely to result in undesired clinical outcomes, such as QT interval prolongation, is paramount. This is especially important in resource limited settings where availability of therapeutic drug monitoring and laboratory follow-up are inconsistent. Adequate quantification of the increased likelihood of adverse clinical outcomes from multiple drug-drug interactions of the same kind in a single patient is needed to aid clinical decisions in this setting.

## Key findings

The result of this analysis shows that potential drug-drug interactions, ranging from minor to contradicted interactions, in this subset of the population are significant and sometimes unavoidable. Although frequency and type of drug-drug interactions have been previously characterized in various populations, this is to our knowledge the first description of potential drug-drug interactions among hospitalized patients living with HIV and presenting with co-morbidities like meningitis and tuberculosis. The findings will be of particular interest to clinicians in similar settings as this information can inform their monitoring and care of these patients especially given the vulnerabilities they face due to their comorbidities.

## Background

The beneficial effects of medications for acute and chronic management of HIV and comorbidities can be lifesaving, but can also implicitly increase the risk of drug related problems [1]. The complexity of the treatment regimen for HIV patients presenting with suspected meningitis further increases their risk of polypharmacy. Polypharmacy is common among hospitalized adult patients and studies conducted in various developed countries report rates of potential drug-drug interactions ranging from approximately 1 to 66% [2]. Drug-drug interactions (DDIs) contribute significantly to adverse drug events such as drug toxicity and ineffective therapy, and can also lead to increased hospital admissions [3, 4].

Potential drug-drug interactions (pDDIs) involve concomitant administration of two drugs identified as having presumed or known clinically significant consequences. Some potentially interacting drug pairs are still likely to be prescribed in combination with relatively high frequency due to limitations on alternative medication therapy for specified treatment regimens [5, 6]. The incidence of actual DDIs (aDDIs) is consistently lower than that of pDDIs [7, 8]. The clinical outcome of a pDDI is often not well defined because available data to track each potential interaction to its actual clinical manifestation is often inconsistent. However, epidemiological data suggest that the absolute number of patients who do experience some kind of drug interaction-based adverse event is high, especially in the presence of known risk factors like older age or polypharmacy [9]. Hence, recognition of pDDIs is valuable as it creates knowledge that aids clinicians to prevent aDDIs.

Resource-limited settings in sub-Saharan Africa often lack adequate therapeutic drug monitoring and other laboratory follow up to monitor outcomes from potentially interacting drugs. Identification of common pDDIs in this population, in addition to risk factors that can predict the likelihood of an adverse effect is crucial for patient safety. For patients on antiretrovirals (ARVs), pDDIs have been reported in the outpatient settings in low and middle income countries [10, 11]. However, there is currently no data available for pDDIs in hospitalized HIV-infected patients presenting with life threatening co-infections such as meningitis. The aim of this study was to characterize the frequency and types of pDDIs involving HIV patients presenting with suspected meningitis in a hospital setting in Uganda.

## Methods

### Study setting and population

This study was a retrospective review of three HIV-associated cryptococcal meningitis trials that enrolled patients between 2010 and 2017 in Kampala, Uganda. The first trial enrolled antiretroviral (ARV)-naïve patients living with HIV with no previous history of cryptococcal meningitis (NCT01075152, COAT) and randomized participants to initiate antiretroviral therapy (ART) either early (1-week) or late (4–6 weeks) after diagnosis of cryptococcal meningitis [12]. The second trial consisting of an open-label pilot and the third a randomized phase 3 trial, enrolled both ART-naïve and ART-experienced patients living with HIV and followed both those presenting with first episode of cryptococcal meningitis and those with previous history of cryptococcosis (NCT01802385, ASTRO) [13, 14]. In these three trials, ART was generally initiated or switched 1–6 weeks after diagnosis of cryptococcal meningitis. All participants had documented HIV-1 infection. Subjects with no available medication therapy record during their hospital stay were excluded. The original trials that provided the data used for this analysis received written, informed patient consent and were approved by the Mulago Research Ethics committee, the Uganda National Council of Science and Technology and the National Drug Authority.

### Data collection

Drug utilization review for de-identified patients were extracted for the patient’s hospital stay (maximum of 30 days drug record). Patients who were screened but not enrolled for either of the above mentioned original studies, but who had documented drug therapy during their hospital stay were also included in this analysis. A pDDI was recorded if the patient received the interacting drug pair on the same calendar day and were included regardless of drug dose or administration time. Fixed dose combination (FDC) drugs such as tenofovir/lamivudine (TDF/3TC) are considered two individual drugs for the purposes of identifying pDDIs. Extracted data was collated on Java with programming language on IntelliJ IDE®.

### Classification of drug interaction and strength of scientific evidence

IBM Micromedex DRUGDEX® system was used to define the types of pDDIs [15]. The software has been previously validated [16]. Drug interactions were classified into 4 main categories based on the likelihood and severity if the interaction were to occur; (i) contraindicated – the two drugs should not be used concurrently; (ii) major – the interaction may be life-threatening and/or require medical intervention to minimize or prevent serious adverse effects; (iii) moderate – the interaction may result in exacerbation of the patient’s condition and/or require an alteration therapy; and (iv) minor – the interactions have limited clinical effects that may include an increase in the frequency or severity of the adverse effects, but generally would not require a major alteration in therapy.

Specifically for interactions involving an antiretroviral drug, clinical significance was further assessed and compared to recommendations from Micromedex using the online University of Liverpool HIV drug interaction checker [17]. This database has been curated for HIV drugs and provides guidelines for management of clinically significant HIV drug interactions. This database is continuously updated as new information becomes available and clinicians can search this database either by individual drug name or by drug class.

### Statistical analysis

Descriptive statistics were used and presented as means and percentages unless otherwise specified. We analyzed continuous variables with t test or Mann-Whitney U test, and we analyzed categorical variables with chi-square. We defined significance (*p < .05*) in univariate analysis. We conducted our statistical analysis using RStudio® Version 1.1.456 [18].

## Results

Medication administration records were available for 1074 patients presenting with suspected meningitis and screened. The median age of participants was 35 years (IQR, 30, 41 years). Fifty-seven percent (617/1074) were males. There was an average (range) of 8 (1–27) drugs prescribed per patient during the 30-day window. Table 1 compares demographic characteristics of patients who had at least one pDDI versus those who did not have any. Significant differences in the two groups were observed in the number of medications administered, baseline CD4, initial diagnosis of tuberculosis and cryptococcal meningitis. Outcome characteristics such as days of stay in hospital and total number of administered drugs were also significantly different between groups. (Table 1) No difference was noted between the two groups for age, gender and diagnosis of malaria.

**Table 1:** Comparison of characteristics of patients with and without at least one potential drug-drug interaction

	One or more pDDI in study period ***n*** = 959	No pDDI in study period ***n*** = 115	***p*** value*
**Demographics**			
**Age**			0.477
**17–30**	316 (32.9)	42 (36.5)	
**31–40**	403 (42.0)	48 (41.7)	
**41–50**	203 (21.2)	20 (17.4)	
**51+**	37 (3.9)	5 (4.3)	
**Gender**			0.070
**Male**	560 (58.4)	57 (49.6)	
**Female**	399 (41.6)	58 (50.4)	
**Baseline diagnostics and investigations**			
**CD4 (cells/mm**^**3**^**)**	18 (7–55)	35 (16–209)	0.040
**Diagnosis of cryptococcal meningitis**			<.001
**No**	364 (38.0)	105 (91.3)	
**Yes**	595 (62.0)	10 (8.7)	
**Diagnosis of tuberculosis**			0.049
**No**	852 (88.8)	109 (94.8)	
**Yes**	107 (11.2)	6 (5.2)	
**Diagnosis of malaria**			0.209
**No**	946 (98.6)	115 (100)	
**Yes**	13 (1.4)	0	
**GCS on admission**			0.003
**15**	588 (61.4)	54 (47)	
**< 15**	369 (38.6)	61 (53)	
**CSF QCC (log**_**10**_**CFU/mL)**	4.58 (2.9–5.4)	0 (0–6.6)	0.053
**In-patient data**			
**Number of administered drugs**	7 (5–9)	3 (2–4)	<.001
**Days of stay in hospital**	16 (11–19)	8 (4.5–13)	<.001

Number of administered drugs, days in hospital, CD4, CSF QCC = median (25th -75th percentile)

Category of age, gender, diagnoses and GCS

*CSF* Cerebral Spinal Fluid, *QCC* Quantitative Cryptococcal Culture, *CFU* Colony Forming Units – for patients presenting with suspected cryptococcal meningitis and for whom CSF culture was collected and quantified (*N* = 947 with at least one DDI; *N* = 112 with no DDI)

* t test of means for continuous variables and chi-square for categorical variables with significance at *p* < .05

Of the total patients included in this sub-analysis, at least one pDDI were in 959 (overall prevalence = 89.3%). In total, 278 unique interacting drug combinations were identified resulting in 4582 pDDI events. (Supplemental Table 1) For all patients included in this study, there was a mean frequency of 4.27 pDDIs per patient with an overall range of 0–23 pDDIs. The majority (60%) of patients had 1–4 pDDIs, 31.3% had 5–10 pDDIs and 8.7% had > 10 pDDIs. For severity classification of the 4582 events, 11.3% were contraindicated, 66.4% were major, 17.4% were moderate and 5% were minor pDDIs. Table 2 summarizes the most frequently occurring pDDIs for each level of severity along with level of scientific evidence and proposed summary of expected clinical effects. Eighty percent of the top occurring contraindicated drugs were classified as having the potential to cause QT interval prolongation (Table 2). No differences with respect to incidence of pDDIs was observed between the three study timelines included.

**Table 2:** Most occurring potential drug-drug interaction for each level of severity

Severity^**a**^	Drug 1	Drug 2	% pDDI overall^**b**^	Level of evidence^**c**^	Proposed effect summary
**Contraindicated**					
	Fluconazole	Ondansetron	3.6	Fair	Risk of QT interval prolongation
	Fluconazole	Haloperidol	2.8	Fair	Increased haloperidol exposure, risk of QT interval prolongation
	Fluconazole	Ritonavir	1.1	Fair	Increased ritonavir exposure, risk of QT interval prolongation
	Artane	Potassium (oral)	0.8	Fair	Gastrointestinal lesions
	Fluconazole	Atazanavir	0.8	Fair	Increased atazanavir exposure, risk of QT interval prolongation
	Fluconazole	Artemether- lumefantrine	0.7	Fair	Risk of QT interval prolongation
	Dihydroartemisinin-piperaquine	Efavirenz	0.2	Fair	Risk of QT interval prolongation
	Fluconazole	Dihydroartemisinin-piperaquine	0.2	Fair	Risk of QT interval prolongation
	Haloperidol	Metoclopramide	0.2	Fair	Increased extrapyramidal reactions and neuroleptic malignant syndrome
	Fluconazole	Quinine	0.1	Fair	Increased quinine levels, risk of QT interval prolongation
**Major**					
	Co-trimoxazole	Fluconazole	18	Fair	Cardiotoxicity (QT prolongation, torsades)
	Efavirenz	Fluconazole	8.6	Fair	Risk of QT interval prolongation
	Codeine	Fluconazole	4.8	Fair	Increased codeine concentration
	Isoniazid	Rifampin	4.5	Good	Hepatotoxicity
	Co-trimoxazole	Haloperidol	2.4	Fair	Cardiotoxicity (QT prolongation, torsades)
	Pyrazinamide	Rifampin	2.4	Good	Hepatotoxicity
	Fluconazole	Metronidazole	2.3	Fair	Risk of QT interval prolongation and arrhythmias
	Efavirenz	Ondansetron	1.3	Fair	QT interval prolongation
	Codeine	Efavirenz	1.1	Fair	Decreased codeine efficacy
	Codeine	Metoclopramide	1.1	Fair	Increased CNS depression
	Azithromycin	Fluconazole	1.1	Fair	Risk of QT interval prolongation
	Ciprofloxacin	Fluconazole	1.1	Fair	Risk of QT interval prolongation
	Fluconazole	Tramadol	0.9	Fair	Increased tramadol exposure and increased risk of respiratory depression
	Acetaminophen	Isoniazid	0.9	Excellent	Hepatotoxicity
	Efavirenz	Rifampin	0.9	Fair	Decreased serum efavirenz concentration
**Moderate**					
	Fluconazole	Zidovudine	4.2	Good	Increased zidovudine serum concentration
	Fluconazole	Rifampin	2.2	Excellent	Decreased fluconazole serum concentration
	Artane	Haloperidol	1.2	Good	Excessive anticholinergic effects
	Acetaminophen	Zidovudine	0.9	Good	Hepatotoxicity (acetaminophen driven)
**Minor**					
	Co-trimoxazole	Zidovudine	3.6	Good	Increased zidovudine serum concentration

^a^ Severity classification for clinical purposes per IBM Micromedex DRUGDEX® database definitions

^b^ Percent of overall pDDI for study, % reported as (n/ 4582 total pDDI events) * 100

^c^ Strength of scientific data for the interaction per IBM Micromedex DRUGDEX® database; (i) excellent – clearly documented well controlled studies support the interaction; (ii) good – studies strongly suggest that interaction exists however there are not well controlled studies; (iii) fair – available evidence is poor but clinicians suspect the interaction exists based on pharmacology or the available evidence is good for a pharmacologically similar drug; and (iv) unknown – interaction documentation is unknown

**Fig. 1:**
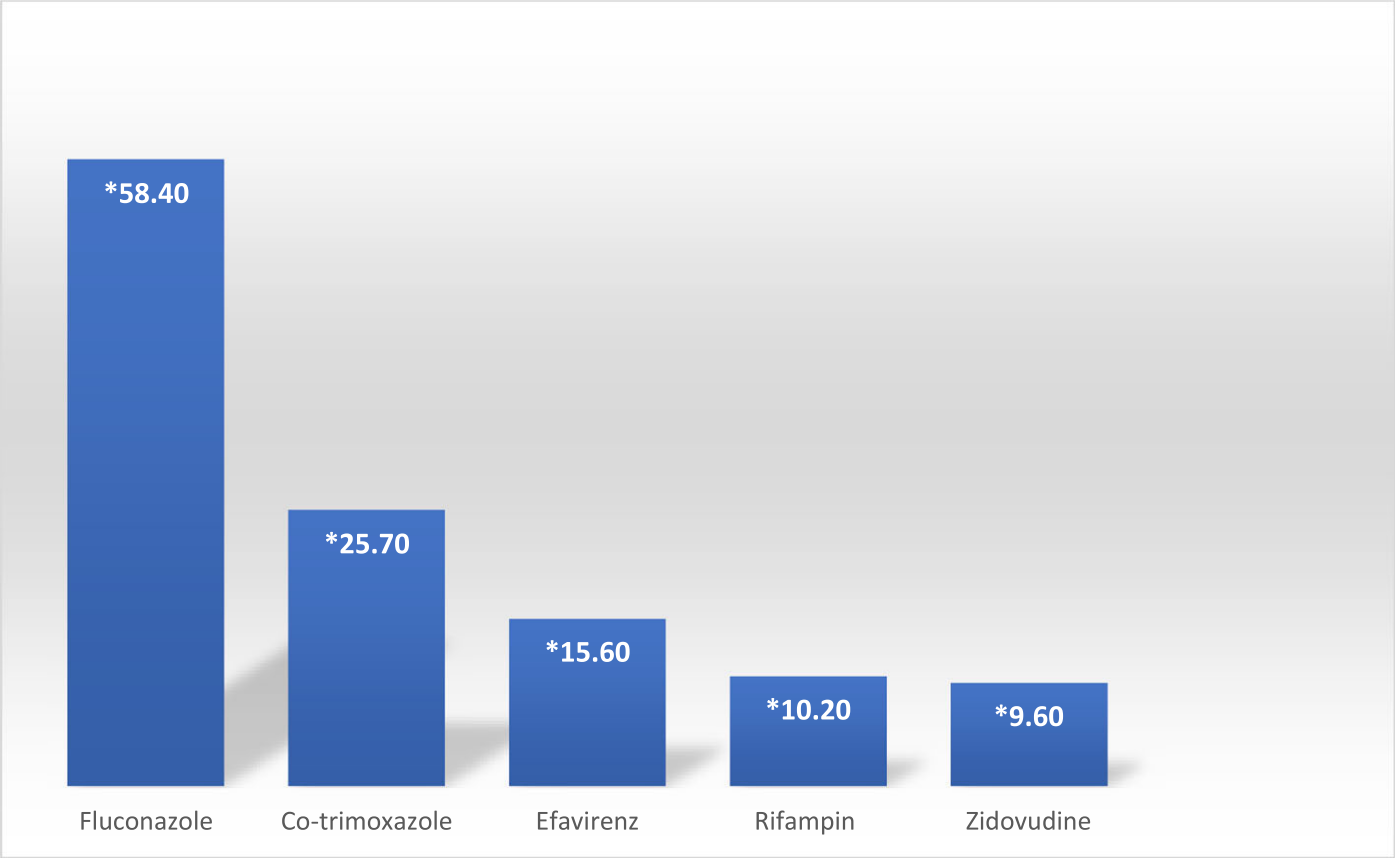
Drugs most implicated for any potential drug-drug interaction by percentage of occurrence in overall analysis. *Percentage calculated by dividing number of interactions involving identified drug with the total number of observed pDDI events (4582 total). Note that some of the identified drugs also interact with each other

Among all pDDIs identified, the most prevalent drugs implicated are represented in Fig. 1**.** Fluconazole was the most prevalent drug interacting with other drugs at 58.4% of overall pDDI events. Some of the other more prevalent drugs also interacted with each other. For example, the most common interaction observed in this study is a major interaction between fluconazole and co-trimoxazole (18.3% of the 4582 events) (Table 2). Potential effects of concurrent use of this drug pair per literature is an increased risk of cardiotoxicity including QT prolongation, *torsades de pointes* and cardiac arrest. Any interaction involving two individual medications that can prolong the QT interval is classified as a major or contraindicated interaction on Micromedex.

Even though the design of the original trial studies excluded ART-experienced patients in COAT and ART initiation was delayed for half of the randomized patients in all three studies, interactions involving at least one antiretroviral were still prevalent at 30.9% of overall pDDI events (regardless of severity). Antiretrovirals were involved in 20.9% of contraindicated pDDIs and 27% of major pDDIs. Table 3 summarizes frequently occurring contraindicated and major pDDIs involving an antiretroviral drug. Additionally, this table takes into consideration clinical recommendations for approaching each drug combination as identified by the University of Liverpool HIV drug interaction website. For comparison, interactions involving antiretrovirals as identified by IBM Micromedex DRUGDEX® system are included in Supplemental Table 1.

**Table 3:** Comparison of recommendation from drug interaction checkers for frequently occurring antiretroviral (ARV)- associated contraindicated and major potential drug-drug interactions

Drug 1	Drug 2	Proposed effect summary – Micromedex^**a**^	Scientific evidence; clinical significance – Liverpool^**b**^	Recommended adjustment- Liverpool^**b**^
Atazanavir	Fluconazole	Increased atazanavir exposure and risk of QT interval prolongation	Low; no interaction expected	No prior dose adjustment necessary
Efavirenz	Codeine	Decreased opioid efficacy	Very low; potential weak interaction	No prior dose adjustment but monitor analgesic effect
Efavirenz	Fluconazole	Increased risk of QT interval prolongation	Moderate; no interaction expected	No dose adjustment necessary
Efavirenz	Haloperidol	Decrease in drug 2 exposure; QT interval prolongation	Very low; potential weak interaction	No prior dose adjustment is recommended
Efavirenz	Metronidazole	Increased risk of QT interval prolongation	Very low; no interaction expected	No dose adjustment necessary
Efavirenz	Ondansetron	Increased risk of QT interval prolongation	Very low; no interaction expected	No dose adjustment necessary
Efavirenz	Rifampin	Decrease in drug 1 serum concentration	High; potential weak interaction	No dose adjustment recommended in patients weighing < 50 kg
Ritonavir	Fluconazole	Increased ritonavir exposure and risk of QT interval prolongation	Very low; no interaction expected	No prior dose adjustment recommended
Tenofovir-DF	Atazanavir	Increase in drug 1 exposure and decrease in drug 2 exposure	Moderate; do not co-administer	Avoid co-administration, if necessary, monitor for adverse effects
Zidovudine	Pyrazinamide	Decrease drug 2 serum concentration	Very low; no interaction expected	No dose adjustment necessary

^a^Effect summary from IBM Micromedex DRUGDEX® system

^b^Clinical recommendations as provided in the University of Liverpool HIV drug interaction website

## Discussion

The present study shows that the overall prevalence of pDDIs in this population is high at 89.3%. Our current study findings are affected by the class of medications that are required for this patient population due to the unavailability of alternate drugs for management of complications of cryptococcal meningitis. For example, fluconazole and cotrimoxazole accounted for 18% of overall pDDIs events but both of these agents are warranted in this population. The incidence rate observed in this study is, however, still congruent with published literature on pDDIs in developing countries. Overall prevalence of pDDIs in reports from hospital settings in Uganda, Ethiopia, Pakistan and Iran range from 23 to 86% [19–24]. In our study, moderate and minor interactions that may require no changes to drug regimen represented ~ 25% of the overall pDDIs observed. A further look at studies considering only Clinically Significant Drug Interactions (CSDI), defined as drug interactions that require a dosage adjustment or are contraindicated due to high potential for clinical adverse effects, report lower overall prevalence. CSDI incidence observed in Kenya and Uganda (both in outpatient settings) range from 18.8 to 33.5% [10, 11]. In the United States and Europe, studies have shown that pDDIs may affect 40–65% of all hospitalized patients [25, 26].

While there is a relatively high prevalence of pDDI in reporting studies, actual drug-drug interactions (aDDIs) that lead to patient harm are often reported as being lower [8]. However, the studies that show lower rates of aDDIs have mostly been done in high-income countries with reliable access to monitoring [8, 9]. While lower aDDIs can be expected in resource limited settings as well, it is not clear to what extent. Identifying pDDIs continues to reinforce measures to keep aDDI low in these settings. Other DDI studies in the literature concur with our results that identify a significant difference in likelihood of having a pDDI for patients with higher total number of medications, extended hospital stay, and high risk diagnoses [4, 7, 19, 27].

The patient population in this present study is unique as all those included had a confirmed HIV diagnosis. Hence, the pDDI observed warrant further discussion. In contrast to the current study, potential drug-drug interactions reported in a general referral hospital in Uganda identified a different set of drug pairs as the most frequently occurring interactions. Lubinga et al identified non-steroidal anti-inflammatory drugs (NSAIDS) in combination with oral corticosteroids; loop diuretics with ACE-inhibitors; and loop diuretics with NSAIDS as the top three occurring potential drug interactions in their Ugandan cohort [19]. This is different in our study population where we have observed that medications used in the management of chronic HIV comorbidities, ARVs and medications used for acute opportunistic infections such as cryptococcal meningitis or tuberculosis presented as the most implicated pDDI drugs. Furthermore, this difference is expected due to treatment guidelines that recommend specifically avoiding the use of NSAIDS in our very ill population with a high risk of concurrent renal impairment, gastrointestinal bleeding, and anemia.

The most frequently occurring pDDI in this study is considered a major interaction between fluconazole and co-trimoxazole. These two agents are used often in patients living with HIV in sub-Saharan Africa and is also a preferred combination for certain disease states. Fluconazole at high doses is used for induction therapy for acute management of cryptococcal meningitis and at lower doses for consolidation therapy. With unavailability of flucytosine, which is now the recommended first line agent for treatment of cryptococcal meningitis, high dose fluconazole remains mainstay induction and consolidation therapy in many resource limited settings. Co-trimoxazole is the preferred drug for both the treatment and prophylaxis of pneumocystis pneumonia in patients living with HIV. Individually, these two medications can cause QT interval prolongation and hence a combination is presumed to increase this risk. QT interval prolongation is an electrocardiographic (ECG) abnormality that has the potential to cause severe arrhythmias including *torsade de pointes (*TdP*)* and ventricular fibrillation. The extent of QT interval prolongation that is expected and further translation to TdP has not been specifically quantified for this drug combination but there is pharmacological speculation that this combination increases risk [15, 28]. Often the risk of QT interval prolongation can be mitigated in high resource settings if the patient is monitored for cardiac abnormalities during the induction phase with high dose fluconazole. In low resource settings however, continuous cardiac monitoring is often not feasible. With little prospective studies available to offer real world translation of the relative risk of this combination, it is often used with no adjustment to dosage. This practice will likely continue unless further research becomes available which suggests changes should be made.

Some studies have described a higher prevalence of QT interval prolongation in HIV positive patients when compared to HIV negative patients [29–31]. In addition to this baseline increase, there is additional risk due to numerous drugs that are administered to HIV infected patients presenting to acute care settings. Arizona Center for Education and Research on Therapeutics (AZCERT) is an evidence based classification system that provides additional clinical guidance to clinicians for evaluating QT prolonging drugs. Drugs with QT prolonging properties are classified to one of four classes - “known risk”, “possible risk”, “conditional risk” (under specific clinical conditions) and “special risk” (in patients with congenital Long QT syndrome) [32]. Drugs in the “known risk” category have sufficient evidence to show that they can cause QT prolongation and are also associated with TdP even at recommended doses while drugs in the “possible risk” category could potentially cause QT interval prolongation but lack evidence of association with TdP at recommended doses. Mied et al. noted in their pharmacological model, that drugs in the “known risk” category are associated with multiplicative effects when combined. A phenomenon not observed when drugs in the other categories are combined [33]. In the second cryptococcal cohort used for this sub analysis, electrocardiogram QT intervals were measured in 53 patients at baseline, day 7, and day 14 of therapy, because a higher dose of sertraline was administered to these patients. The results showed that QT intervals actually decreased over time [34]. It is interesting to note that sertraline is in the “conditional risk” category for QT prolongation. Hence, it might be prudent for clinicians to become more familiar with drugs in the “known risk” category or have access to tools that can easily identify these drugs. In our study, prevalent drugs in the “known risk” category include fluconazole, ondansetron, levofloxacin, erythromycin, ciprofloxacin, azithromycin, and haloperidol [32].

Treatment of other comorbidities such as malaria and tuberculosis (TB) also contributed to the pDDIs. For instance, pDDI of hepatotoxicity was identified with standard tuberculosis medicines of rifampin, isoniazid and pyrazinamide [35]. Liver function tests are a standard to monitor for potential toxicity from these commonly prescribed combination that is part of first-line regimens for tuberculosis. Guideline recommends that clinicians perform liver function tests for those with symptoms of hepatotoxicity [36]. Furthermore, hepatotoxicity is also a concern when isoniazid is combined with high doses of acetaminophen (> 4 g/day) and thus this combination should be used cautiously [37]. Management of patients admitted with malaria also requires caution as there are potential contraindicated and major interactions with anti-malarials. Artemether/lumefantrine and dihydroartemisinin-piperaquine were the most commonly implicated anti-malarials [38]. In combination with efavirenz, there is potential for reduced antimalarial efficacy and hence caution is advised with these combinations.

Other important pDDI events that were observed in this study includes the contraindicated interaction between artane (trihexyphenidyl or benzhexol) and oral potassium potentially causing gastrointestinal lesions due to GI arrest of the potassium tablets [39]. When feasible this combination should be avoided. The package insert for Klor-con® (potassium chloride extended release tablets) warns against the combination of all solid oral dosage forms of potassium with anticholinergic agents. Potential central nervous system (CNS) adverse effects could also occur with specific drug pairs including CNS depression due to increases in opioid concentrations, or on the flip side, opioid withdrawal symptoms due to decreased opioid efficacy [15, 17]. Metoclopramide in combination with haloperidol or sertraline is also classified as a contraindicated pDDI that could lead to extrapyramidal effects and possible neuroleptic malignant syndrome [40].

The University of Liverpool HIV drug interaction website provides clinicians with recommendations for necessary dose adjustments when potentially interacting drugs involving ARVs are combined. Although the IBM Micromedex DRUGDEX® system has been validated to provide accurate information, disagreements still persist among drug interactions checkers [41]. Hence, consulting a database curated specifically for HIV drug interactions was done to provide additional information for the management of various ARV-associated pDDIs observed in the present study. Atazanavir and tenofovir-DF, for example, is one drug combination that should be avoided but if it is absolutely necessary to combine, Atazanavir should be boosted and monitoring of renal function will be needed. Of interest, the Liverpool drug interaction website does not identify interactions that could lead to QT interval prolongation in the same way that IBM Micromedex DRUGDEX® does. Hence, many of the contraindicated and major interactions identified on Micromedex with the potential to cause QT interval prolongation are classified as likely having no potential interaction or needing additional monitoring per Liverpool interaction website. This disagreement is likely due to the focus on CYP-based interactions by the Liverpool interaction website. This dissonance makes it difficult for clinicians to solely rely on just one interaction checker. It, however, creates an opportunity for further collaboration between drug interaction database creators. It is our observation that for chronic monitoring of patients living with HIV, the Liverpool drug interaction checker provides more nuanced information as it is specialized towards specific population(s). However, when patients present to acute settings and require other non-ARV drugs, IBM Micromedex® drug interaction checker still offers valuable drug information that clinicians can use in determining drug-drug interactions that may require additional caution.

Overall, there were only a few potentially life-threatening risks seen among all pDDIs. Many of the drug combinations are essential for treatment and are recommended by clinical guidelines. However, given the unique study setting, the breadth of knowledge from this study could potentially direct future prospective case studies that analyze these identified drug interactions to better document the extent of laboratory monitoring that should be implemented. Furthermore, about 40% of the patients in this study had five or more pDDI. Hence, identifying the overall prevalence of certain drug combinations and the likelihood of multiplicative increases in adverse effects can help inform clinicians. Inclusion of a pharmacist in a patient’s care team have also been shown to aid in identification and reduction of pDDI events [42].

There were strengths to this analysis. This study was unique from previous DDI reports in that we reported prevalence of pDDIs in an acute setting for patients living with HIV and presenting with suspected meningitis. The information obtained from this large study sample also spanned various years (2010 to 2017) and a spectrum of practitioners and prescribing patterns. While this study only looked at a 30-day window for each patient, the variability of the two trial studies adds a strength to data generalization to other settings managing patients in this population. There were also limitations to our analysis. Since pDDI were only assessed as drugs that were co-administered on the same calendar day rather than at specific times during the day, there could have been underestimating or overestimating of concurrent drug exposure. Drugs with a short-life that were administered in the morning could have cleared from the patients system prior to an interacting drug administered later at night. On the flip side, drugs with longer half-life or undergoing other pharmacodynamic processes could still have an effect on an interacting drug beyond the 24 h window. Additionally, the pDDIs were classified regardless of dose adjustments that may or may not have happened. In line with this, the study mainly focused on theoretical drug-drug interactions. Since the data source did not consistently match immediate results of laboratory tests and other mortality defining clinical measurements, there was no way to evaluate true clinical significance or mortality outcomes of pDDI exposure. There may have also been selection bias because the analysis only included patients from the original studies with available drug information, although the percentage of excluded patients were low (< 5%).

## Conclusions

Our findings suggest that the prevalence of potential drug-drug interactions as classified by the IBM Micromedex DRUGDEX® system in our study population is high. A closer look at these drug interactions suggest that only a few present with significant immediate life threatening risks such as CNS depression. However, understanding the drug pairs that could lead to undesired outcomes such as increases or decreases in drug concentrations leading to adverse toxic events or loss of therapeutic efficacy is important. Understanding the extent of unwanted drug effects such as GI lesions, extrapyramidal reactions, hepatotoxicity, QT interval prolongation and other cardiac toxicities is paramount especially while working in resource limited settings like Uganda. We advocate for additional research into dose adjustments necessary for some of the identified pDDIs, as well as further documentation of potential harm and possibly mortality implications from multiple drug-drug interactions of the same effect in a single patient, as these could provide better guidance to clinicians.

## Supplementary information

**Additional file 1: Table S1**. Complete list of all observed potential drug-drug interactions (pDDIs).

## Data Availability

Data available from corresponding author upon reasonable request.

## Abbreviations

3TC: Lamivudine
aDDIs: Actual drug-drug interactions
ART: Antiretroviral therapy
ARVs: Antiretrovirals
CNS: Central nervous system
DDIs: Drug-drug interactions
FDC: Fixed-dose combination
GI: Gastrointestinal
HIV: Human immunodeficiency virus
IQR: Interquartile range
NSADs: Non-steroidal anti-inflammatory drugs
pDDIs: Potential drug-drug interactions
TB: Tuberculosis
TDF: Tenofovir disoproxil fumarate
TdP: Torsade de pointes
