# Supplementary material for: Prevalence and nature of potential drug-drug interactions among hospitalized HIV patients presenting with suspected meningitis in Uganda
Source: BMC Infect Dis. 2020 Aug 5;20:572. doi: 10.1186/s12879-020-05296-w (PMC7405463; doi:10.1186/s12879-020-05296-w)
Supplement: Supplementary file 1 — Additional file 1: Table S1. Complete list of all observed potential drug-drug interactions (pDDIs). [file 12879_2020_5296_MOESM1_ESM.docx]

**SUPPLEMENTAL TABLE 1**

| **Drug combination** | **Severity per IBM Micromedex** |
| --- | --- |
| **AMITRIPTYLINE & METOCLOPRAMIDE** | **Contraindicated** |
| **ARTANE & POTASSIUM** | **Contraindicated** |
| **ARTEMETHER & FLUCONAZOLE** | **Contraindicated** |
| **ARTEMETHER & RIFAMPICIN** | **Contraindicated** |
| **ATAZANAVIR & RANITIDINE** | **Contraindicated** |
| **ATAZANAVIR & RIFAMPICIN** | **Contraindicated** |
| **CARBAMAZEPINE & EFAVIRENZ** | **Contraindicated** |
| **CHLOPROMAZINE & METOCLOPRAMIDE** | **Contraindicated** |
| **DUOCOTEXCIN & EFAVIRENZ** | **Contraindicated** |
| **DUOCOTEXCIN & FLUCONAZOLE** | **Contraindicated** |
| **DUOCOTEXCIN & ONDANSETRON** | **Contraindicated** |
| **EFAVIRENZ & RITONAVIR** | **Contraindicated** |
| **ERYTHROMYCIN & FLUCONAZOLE** | **Contraindicated** |
| **FLUCONAZOLE & HALOPERIDOL** | **Contraindicated** |
| **FLUCONAZOLE & QUININE** | **Contraindicated** |
| **HALOPERIDOL & METOCLOPRAMIDE** | **Contraindicated** |
| **LOPINAVIR & RIFAMPICIN** | **Contraindicated** |
| **METOCLOPRAMIDE & SERTRALINE** | **Contraindicated** |
| **FLUCONAZOLE & ATAZANAVIR** | **Contraindicated** |
| **FLUCONAZOLE & DOMPERIDONE** | **Contraindicated** |
| **FLUCONAZOLE & KYTRIL** | **Contraindicated** |
| **FLUCONAZOLE & ONDANSETRON** | **Contraindicated** |
| **FLUCONAZOLE & RITONAVIR** | **Contraindicated** |
| **RIFAMPICIN & RITONAVIR** | **Contraindicated** |
| **ACETAMINOPHEN & ISONIAZID** | **Major** |
| **AMITRIPTYLINE & AZITHROMYCIN** | **Major** |
| **AMITRIPTYLINE & CODEINE** | **Major** |
| **AMITRIPTYLINE & EFAVIRENZ** | **Major** |
| **AMITRIPTYLINE & FLUCONAZOLE** | **Major** |
| **AMITRIPTYLINE & ONDANSETRON** | **Major** |
| **AMITRIPTYLINE & TRIMETHOPRIM-SULFAMETHOXAZOLE** | **Major** |
| **AMOXICILLIN & DOXYCYCLINE** | **Major** |
| **ARTANE & METOCLOPRAMIDE** | **Major** |
| **ARTEMETHER & AZITHROMYCIN** | **Major** |
| **ARTEMETHER & EFAVIRENZ** | **Major** |
| **ARTEMETHER & HALOPERIDOL** | **Major** |
| **ARTEMETHER & METRONIDAZOLE** | **Major** |
| **ARTEMETHER & NEVIRAPINE** | **Major** |
| **ARTEMETHER & ONDANSETRON** | **Major** |
| **ARTEMETHER & RITONAVIR** | **Major** |
| **ASPIRIN & CLEXANE** | **Major** |
| **ASPIRIN & TENOFOVIR** | **Major** |
| **ATAZANAVIR & CODEINE** | **Major** |
| **ATAZANAVIR & EFAVIRENZ** | **Major** |
| **ATAZANAVIR & METRONIDAZOLE** | **Major** |
| **ATAZANAVIR & OMEPRAZLE** | **Major** |
| **ATAZANAVIR & ONDANSETRON** | **Major** |
| **ATAZANAVIR & TENOFOVIR** | **Major** |
| **AZITHROMYCIN & EFAVIRENZ** | **Major** |
| **AZITHROMYCIN & FLUCONAZOLE** | **Major** |
| **AZITHROMYCIN & HALOPERIDOL** | **Major** |
| **AZITHROMYCIN & KYTRIL** | **Major** |
| **AZITHROMYCIN & LEVOFLOXACIN** | **Major** |
| **AZITHROMYCIN & METRONIDAZOLE** | **Major** |
| **AZITHROMYCIN & ONDANSETRON** | **Major** |
| **CAPTOPRIL & POTASSIUM** | **Major** |
| **CAPTOPRIL & TRIMETHOPRIM-SULFAMETHOXAZOLE** | **Major** |
| **CARBAMAZEPINE & CODEINE** | **Major** |
| **CARBAMAZEPINE & DUOCOTEXCIN** | **Major** |
| **CARBAMAZEPINE & FLUCONAZOLE** | **Major** |
| **CARBAMAZEPINE & ISONIAZID** | **Major** |
| **CARBAMAZEPINE & PHENYTOIN** | **Major** |
| **CARBAMAZEPINE & QUININE** | **Major** |
| **CHLOPROMAZINE & CODEINE** | **Major** |
| **CHLOPROMAZINE & EFAVIRENZ** | **Major** |
| **CHLOPROMAZINE & FLUCONAZOLE** | **Major** |
| **CHLOPROMAZINE & HALOPERIDOL** | **Major** |
| **CHLOPROMAZINE & KETOCONAZOLE** | **Major** |
| **CHLOPROMAZINE & METRONIDAZOLE** | **Major** |
| **CHLOPROMAZINE & MORPHINE** | **Major** |
| **CHLOPROMAZINE & ONDANSETRON** | **Major** |
| **CHLOPROMAZINE & TRAMADOL** | **Major** |
| **CHLOPROMAZINE & TRIMETHOPRIM-SULFAMETHOXAZOLE** | **Major** |
| **CIPROFLOXACIN & CODEINE** | **Major** |
| **CIPROFLOXACIN & EFAVIRENZ** | **Major** |
| **CIPROFLOXACIN & ERYTHROMYCIN** | **Major** |
| **CIPROFLOXACIN & FLUCONAZOLE** | **Major** |
| **CIPROFLOXACIN & HALOPERIDOL** | **Major** |
| **CIPROFLOXACIN & ONDANSETRON** | **Major** |
| **CIPROFLOXACIN & PREDNISOLONE** | **Major** |
| **CIPROFLOXACIN & TRAMADOL** | **Major** |
| **CLEXANE & WARFARIN** | **Major** |
| **CLONAZEPAM & CODEINE** | **Major** |
| **CLONAZEPAM & METOCLOPRAMIDE** | **Major** |
| **CODEINE & EFAVIRENZ** | **Major** |
| **CODEINE & ERYTHROMYCIN** | **Major** |
| **CODEINE & FLUCONAZOLE** | **Major** |
| **CODEINE & HALOPERIDOL** | **Major** |
| **CODEINE & METOCLOPRAMIDE** | **Major** |
| **CODEINE & MORPHINE** | **Major** |
| **CODEINE & ONDANSETRON** | **Major** |
| **CODEINE & PHENYTOIN** | **Major** |
| **CODEINE & RITONAVIR** | **Major** |
| **CODEINE & SERTRALINE** | **Major** |
| **CODEINE & TRAMADOL** | **Major** |
| **DEXAMETHASONE & DICLOFENAC** | **Major** |
| **DEXAMETHASONE & EFAVIRENZ** | **Major** |
| **DEXAMETHASONE & VINCRISTINE** | **Major** |
| **DIAZEPAM & METOCLOPRAMIDE** | **Major** |
| **DICLOFENAC & TENOFOVIR** | **Major** |
| **DOXYCYCLINE & PIPERACILLIN.TAZOBACTAM** | **Major** |
| **DUOCOTEXCIN & ISONIAZID** | **Major** |
| **DUOCOTEXCIN & RIFAMPICIN** | **Major** |
| **EFAVIRENZ & ERYTHROMYCIN** | **Major** |
| **EFAVIRENZ & FLUCONAZOLE** | **Major** |
| **EFAVIRENZ & HALOPERIDOL** | **Major** |
| **EFAVIRENZ & METRONIDAZOLE** | **Major** |
| **EFAVIRENZ & NEVIRAPINE** | **Major** |
| **EFAVIRENZ & ONDANSETRON** | **Major** |
| **EFAVIRENZ & PETHIDINE** | **Major** |
| **EFAVIRENZ & PROCHLORPERAZINE** | **Major** |
| **EFAVIRENZ & RIFAMPICIN** | **Major** |
| **EFAVIRENZ & RISPERIDONE** | **Major** |
| **EFAVIRENZ & SERTRALINE** | **Major** |
| **EFAVIRENZ & TRAMADOL** | **Major** |
| **EFAVIRENZ & WARFARIN** | **Major** |
| **ENALAPRIL & MORPHINE** | **Major** |
| **ENALAPRIL & POTASSIUM** | **Major** |
| **ENALAPRIL & TRIMETHOPRIM-SULFAMETHOXAZOLE** | **Major** |
| **ERTAPENEM & VALPROIC ACID** | **Major** |
| **ERYTHROMYCIN & TRIMETHOPRIM-SULFAMETHOXAZOLE** | **Major** |
| **FLUCONAZOLE & LEVOFLOXACIN** | **Major** |
| **FLUCONAZOLE & METRONIDAZOLE** | **Major** |
| **FLUCONAZOLE & PHENOBARBITAL** | **Major** |
| **FLUCONAZOLE & PROCHLORPERAZINE** | **Major** |
| **FLUCONAZOLE & PROMETHAZINE** | **Major** |
| **FLUCONAZOLE & TRAMADOL** | **Major** |
| **FLUCONAZOLE & TRIMETHOPRIM-SULFAMETHOXAZOLE** | **Major** |
| **FLUCONAZOLE & WARFARIN** | **Major** |
| **GENTAMICIN & VANCOMYCIN** | **Major** |
| **HALOPERIDOL & LOPINAVIR** | **Major** |
| **HALOPERIDOL & METRONIDAZOLE** | **Major** |
| **HALOPERIDOL & MORPHINE** | **Major** |
| **HALOPERIDOL & ONDANSETRON** | **Major** |
| **HALOPERIDOL & SERTRALINE** | **Major** |
| **HALOPERIDOL & TRAMADOL** | **Major** |
| **HALOPERIDOL & TRIMETHOPRIM-SULFAMETHOXAZOLE** | **Major** |
| **IBUPROFEN & PREDNISONE** | **Major** |
| **IBUPROFEN & TENOFOVIR** | **Major** |
| **ISONIAZID & RIFAMPICIN** | **Major** |
| **KETOCONAZOLE & PHENYTOIN** | **Major** |
| **KYTRIL & TRAMADOL** | **Major** |
| **LEVOFLOXACIN & METRONIDAZOLE** | **Major** |
| **LEVOFLOXACIN & ONDANSETRON** | **Major** |
| **LEVOFLOXACIN & QUININE** | **Major** |
| **LEVOFLOXACIN & SERTRALINE** | **Major** |
| **LOPINAVIR & METRONIDAZOLE** | **Major** |
| **LOPINAVIR & ONDANSETRON** | **Major** |
| **LOPINAVIR & PREDNISONE** | **Major** |
| **LORAZEPAM & METOCLOPRAMIDE** | **Major** |
| **LORAZEPAM & MORPHINE** | **Major** |
| **LORAZEPAM & TRAMADOL** | **Major** |
| **MEROPENEM & VALPROICACID** | **Major** |
| **METOCLOPRAMIDE & MIDAZOLAM** | **Major** |
| **METOCLOPRAMIDE & MORPHINE** | **Major** |
| **METOCLOPRAMIDE & TRAMADOL** | **Major** |
| **METRONIDAZOLE & ONDANSETRON** | **Major** |
| **METRONIDAZOLE & RITONAVIR** | **Major** |
| **METRONIDAZOLE & SERTRALINE** | **Major** |
| **MIDAZOLAM & MORPHINE** | **Major** |
| **MIDAZOLAM & TRAMADOL** | **Major** |
| **MORPHINE & ONDANSETRON** | **Major** |
| **MORPHINE & PETHIDINE** | **Major** |
| **MORPHINE & TRAMADOL** | **Major** |
| **NEVIRAPINE & RIFAMPICIN** | **Major** |
| **OMEPRAZLE & RITONAVIR** | **Major** |
| **ONDANSETRON & RITONAVIR** | **Major** |
| **ONDANSETRON & SERTRALINE** | **Major** |
| **PHENYTOIN & RIFAMPICIN** | **Major** |
| **PIPERACILLIN.TAZOBACTAM & VANCOMYCIN** | **Major** |
| **PIPERACILLIN.TAZOBACTAM & WARFARIN** | **Major** |
| **PREDNISOLONE & RITONAVIR** | **Major** |
| **PREDNISONE & RITONAVIR** | **Major** |
| **PROCHLORPERAZINE & TRAMADOL** | **Major** |
| **PROCHLORPERAZINE & TRIMETHOPRIM-SULFAMETHOXAZOLE** | **Major** |
| **PYRAZINAMIDE & RIFAMPICIN** | **Major** |
| **RIFAMPICIN & TRAMADOL** | **Major** |
| **RISPERIDONE & TRIMETHOPRIM-SULFAMETHOXAZOLE** | **Major** |
| **TRIMETHOPRIM-SULFAMETHOXAZOLE & PYRIMETHAMINE** | **Major** |
| **TRIMETHOPRIM-SULFAMETHOXAZOLE & WARFARIN** | **Major** |
| **ZIDOVUDINE & DAPSONE** | **Major** |
| **ZIDOVUDINE & PYRAZINAMIDE** | **Major** |
| **FLUCONAZOLE & ALPRAZOLAM** | **Major** |
| **FLUCONAZOLE & CHLOPROMAZINE** | **Major** |
| **FLUCONAZOLE & LOPINAVIR** | **Major** |
| **FLUCONAZOLE & OLANZAPINE** | **Major** |
| **FLUCONAZOLE & PETHIDINE** | **Major** |
| **FLUCONAZOLE & RISPERIDONE** | **Major** |
| **FLUCONAZOLE & SERTRALINE** | **Major** |
| **FLUCONAZOLE & VINCRISTINE** | **Major** |
| **FLUCONAZOLE & TOLVAPTAN** | **Major** |
| **ZIDOVUDINE & STAVUDINE** | **Major** |
| **ACETAMINOPHEN & PHENYTOIN** | **Moderate** |
| **ACETAMINOPHEN & WARFARIN** | **Moderate** |
| **ACETAMINOPHEN & ZIDOVUDINE** | **Moderate** |
| **ACYCLOVIR & PHENYTOIN** | **Moderate** |
| **ACYCLOVIR & VALPROICACID** | **Moderate** |
| **ANTACID & POTASSIUM** | **Moderate** |
| **ARTANE & HALOPERIDOL** | **Moderate** |
| **ARTANE & VALPROICACID** | **Moderate** |
| **ASPIRIN & PREDNISOLONE** | **Moderate** |
| **ASPIRIN & VALPROICACID** | **Moderate** |
| **ATAZANAVIR & DAPSONE** | **Moderate** |
| **ATAZANAVIR & MAGNESIUM** | **Moderate** |
| **AZITHROMYCIN & PHENYTOIN** | **Moderate** |
| **CAPTOPRIL & LASIX** | **Moderate** |
| **CARBAMAZEPINE & HALOPERIDOL** | **Moderate** |
| **CHLORAMPHENICOL & RIFAMPICIN** | **Moderate** |
| **CIPROFLOXACIN & PHENYTOIN** | **Moderate** |
| **CLONAZEPAM & FLUCONAZOLE** | **Moderate** |
| **DAPSONE & RIFAMPICIN** | **Moderate** |
| **DEXAMETHASONE & PHENYTOIN** | **Moderate** |
| **DEXAMETHASONE & RIFAMPICIN** | **Moderate** |
| **DIAZEPAM & ISONIAZID** | **Moderate** |
| **DIAZEPAM & RIFAMPICIN** | **Moderate** |
| **DIAZEPAM & RITONAVIR** | **Moderate** |
| **DICLOFENAC & FLUCONAZOLE** | **Moderate** |
| **DICLOFENAC & LEVOFLOXACIN** | **Moderate** |
| **DICLOFENAC & RIFAMPICIN** | **Moderate** |
| **DOXYCYCLINE & MAGNESIUM** | **Moderate** |
| **DOXYCYCLINE & RIFAMPICIN** | **Moderate** |
| **DUOCOTEXCIN & OMEPRAZLE** | **Moderate** |
| **EFAVIRENZ & MEDROXYPROGESTERONE** | **Moderate** |
| **EFAVIRENZ & PHENYTOIN** | **Moderate** |
| **ETHOSUXAMIDE & VALPROICACID** | **Moderate** |
| **FLUCONAZOLE & LORAZEPAM** | **Moderate** |
| **FLUCONAZOLE & MIDAZOLAM** | **Moderate** |
| **FLUCONAZOLE & OMEPRAZLE** | **Moderate** |
| **FLUCONAZOLE & PHENYTOIN** | **Moderate** |
| **FLUCONAZOLE & PREDNISONE** | **Moderate** |
| **FLUCONAZOLE & RIFAMPICIN** | **Moderate** |
| **GENTAMICIN & MAGNESIUM** | **Moderate** |
| **HALOPERIDOL & RIFAMPICIN** | **Moderate** |
| **HYCROCORTISONE & LASIX** | **Moderate** |
| **IBUPROFEN & LEVOFLOXACIN** | **Moderate** |
| **INSULIN & TRIMETHOPRIM-SULFAMETHOXAZOLE** | **Moderate** |
| **IRON & OMEPRAZLE** | **Moderate** |
| **ISONIAZID & PHENYTOIN** | **Moderate** |
| **ISONIAZID & VALPROICACID** | **Moderate** |
| **ISONIAZID & WARFARIN** | **Moderate** |
| **LOPINAVIR & TENOFOVIR** | **Moderate** |
| **LOPINAVIR & VALPROICACID** | **Moderate** |
| **METFORMIN & RIFAMPICIN** | **Moderate** |
| **METRONIDAZOLE & PHENYTOIN** | **Moderate** |
| **MIDAZOLAM & RIFAMPICIN** | **Moderate** |
| **MORPHINE & RIFAMPICIN** | **Moderate** |
| **NEVIRAPINE & WARFARIN** | **Moderate** |
| **OLANZAPINE & VALPROICACID** | **Moderate** |
| **PHENYTOIN & PREDNISOLONE** | **Moderate** |
| **PHENYTOIN & PREDNISONE** | **Moderate** |
| **PHENYTOIN & TRIMETHOPRIM-SULFAMETHOXAZOLE** | **Moderate** |
| **PHENYTOIN & VALPROICACID** | **Moderate** |
| **PREDNISOLONE & RIFAMPICIN** | **Moderate** |
| **PREDNISONE & RIFAMPICIN** | **Moderate** |
| **RIFAMPICIN & SERTRALINE** | **Moderate** |
| **RIFAMPICIN & VALPROICACID** | **Moderate** |
| **RITONAVIR & TENOFOVIR** | **Moderate** |
| **RITONAVIR & VALPROICACID** | **Moderate** |
| **VANCOMYCIN & WARFARIN** | **Moderate** |
| **ZIDOVUDINE & FLUCONAZOLE** | **Moderate** |
| **ZIDOVUDINE & RIFAMPICIN** | **Moderate** |
| **FLUCONAZOLE & RABEPRAZOLE** | **Moderate** |
| **ZIDOVUDINE & VALPROICACID** | **Moderate** |
| **ACYCLOVIR & ZIDOVUDINE** | **Minor** |
| **ALBENDAZOLE & DEXAMETHASONE** | **Minor** |
| **AMPICILIN & GENTAMICIN** | **Minor** |
| **ANTACID & ISONIAZID** | **Minor** |
| **ATENOLOL & MAGNESIUM** | **Minor** |
| **AZITHROMYCIN & MAGNESIUM** | **Minor** |
| **DIAZEPAM & OMEPRAZLE** | **Minor** |
| **DIAZEPAM & TRAMADOL** | **Minor** |
| **GENTAMICIN & PIPERACILLIN.TAZOBACTAM** | **Minor** |
| **IRON & MAGNESIUM** | **Minor** |
| **ISONIAZID & PREDNISOLONE** | **Minor** |
| **ISONIAZID & PREDNISONE** | **Minor** |
| **LASIX & PHENYTOIN** | **Minor** |
| **ZIDOVUDINE & RITONAVIR** | **Minor** |
| **ZIDOVUDINE & TRIMETHOPRIM-SULFAMETHOXAZOLE** | **Minor** |
